# Social Support as a Moderator of the Relationship between Anxiety and Depression: An Empirical Study with Adult Survivors of Wenchuan Earthquake

**DOI:** 10.1371/journal.pone.0079045

**Published:** 2013-10-18

**Authors:** Jiuping Xu, Ying Wei

**Affiliations:** Uncertainty Decision-making Laboratory, Sichuan University, Chengdu, P. R. China; University of Electronic Science and Technology of China, China

## Abstract

**Background:**

On May 12th 2008, an earthquake with a magnitude of 8.0 on the Richter scale struck China, causing a large number of casualties and significant economic losses. By interviewing 2080 survivors of Wenchuan earthquake, the objective of this study is to estimate the role of different types of social support as possible moderating factors between anxiety and depression.

**Methods:**

A stratified random sampling strategy about the cross-sectional study was adopted. The self-rating anxiety scale (SAS), Self-Rating Depression Scale (SDS) and Social Support Rating Scale (SSRS) were used. A total of 2080 adult survivors of the Wenchuan earthquake from 19 damaged countries participated in the survey. Correlation analysis and regression analysis were performed to evaluate the moderating role of social support on the relationship between anxiety and depression.

**Results:**

One year after the Wenchuan earthquake, anxiety and depression were found to be 37.6% and 40.7%, respectively. Demographic characteristics were seen as significant in the cases of depression, except for age (p=0.599), while age and education level were not found to be significant for anxiety. The results showed that social support, especially subjective support could moderate the association between anxiety and depression.

**Conclusions:**

Social support should be particularly focused on female survivors, those of the Han ethnic group, and those with a lower level of education and a lower income. Psychological intervention and care for survivors should focus on those most disoriented by the disaster.

## Introduction

When an unexpected event occurs, people’s behavior is most deeply affected by political, economic, social, and cultural contexts. Epidemiological studies indicate that most adults are exposed to at least one potentially traumatic event during their lifetime [1]. Natural disasters not only cause problems such as property losses, infrastructure damage and resource destruction, but also cause secondary psychological disorders [2-4]. Earthquakes have caused great damage to people’s personal and economic wellbeing, and caused significant psychological trauma to survivors since the twentieth century [5,6]. It has been shown that it is important to pay attention to the mental health of survivors after earthquake.

Depressive symptoms, which have been ranked the world's fourth largest disorder, and are expected become second to heart disease by 2020 [7], have been linked to many risk factors, including, but not limited to life event stress, genetic factors, or somatopathy. However, it should be noted that depression is different from depressive symptoms, as depression is a main or important manifestation of many diseases and not only associated with depressive symptoms. Research on the effect of earthquakes has typically paid attention to the subsequent negative psychological and behavioral changes and stress reactions, with depression and anxiety being found to be two common disorders. There are varying opinions about the relationship between anxiety and depression, which can be summarized as follows: (1) the comorbidity of anxiety disorders and either major depressive disorders or dysthymia; (2) one of the two induces changes that lead to the other; and (3) anxiety has contributed to depression. Tull and Gratz examined the relationship to distinguish participants with clinical levels of depression [8]. Fentz et al. found that there were no differences in the parenting styles between the three diagnostic anxiety groups with depressive symptoms [9], while Hansen et al. indicated that there was a positive relationship between them [10]. Such findings highlight the importance of focusing on the relationship between depression and anxiety. In general, as described above, anxiety can be considered primary and depression considered secondary in the temporal chain of event appraisal.

In the study of mental health problems arising from disaster, some scholars have long advocated paying attention to social factors, with the thought that variations in these factors could reduce, enlarge or alter the effects of the disaster on individual mental health. Hofmann et al. concluded that social anxiety and depression were more or less associated with significant social functioning problems [11]. Social support, as an important social resource for an individual to cope with stressful life events, can buffer life events and psychological symptoms directly as well as indirectly [12,13]. Many studies have examined the relationship between social support and depression, and the findings generally show that social support plays an important role in depression, indicating that people who receive more social support can reduce the risk of developing depressive symptoms. Holohan et al. showed that higher perceptions of social support predicted fewer symptoms of depression [14]. Han et al. studied elderly people and found that an increased risk of depression was associated with a lack of social support in several communities [15]. When considering anxiety, Drageset found that social support did not in itself repress anxiety after interviewing women who had suffered suspected breast cancer [16]. To our knowledge, previous research has investigated how social support relates to anxiety and depression separately, but few have examined the relationship in a single model. With this in mind, in this study, we took social support as a moderator, hypothesizing in an anxiety-depression model that social support has the possibility to mitigate the negative impact of earthquake events on survivors’ wellbeing post-earthquake.

The present study was established in an attempt to further investigate the relationship between anxiety and depression in survivors of the May 12th 2008 Wenchuan earthquake. This earthquake event has been ranked as the most devastating earthquake since the founding of new China, as not only did it destroy local infrastructure and cause large economic losses, but also heavily affected the population. In this paper, special attention is paid to the relationship between anxiety and depression, the prevalence of which was evaluated among survivors to determine the factors which could assist in a reduction in negative influences. On the basis of this conceptualization, this study sought to test the main and interactive effects of anxiety and social support in terms of depression over time. 

## Materials and Methods

### Ethics Statement

Data were analyzed anonymously. The study and procedures were approved by the ethics committee of Sichuan University and written informed consent was obtained from each subject after a full explanation of the study procedures. The investigation was conducted in accordance with the latest version of the Declaration of Helsinki. 

To assure a reasonable distribution of gender, age and place, the inclusion criteria was a high degree of exposure to earthquake, and an experience of the whole earthquake process. A total of 2300 survivors from the Wenchuan earthquake were interviewed, all of whom were direct victims and had had their houses fully or partly collapsed. Some declined the interview as they were afraid of reliving the disaster and wished to avoid talking about the event, so 2080 of the 2300 individuals completed the questionnaire, a response rate of nearly 90.4%. 

### Instruments

Participants completed paper-and-pencil self-report personality questionnaires, covering items on four sets of data, including demographic characteristics, anxiety, depression and social support. 

Demographic characteristics consisted of gender, age, ethnicity, education level and monthly income (see Table 1), which were evaluated for inclusion as potential covariates in the analyses. Gender was coded as 1 (male) and 2 (female). Age was divided into four groups, and in regard to ethnicity, four main ethnicities Han, Tibetan, Hui, and Qiang were included. Education was divided into three levels, and monthly income was split into four groups, from less than 1000 RMB to over 3000 RMB.

**Table 1:** Socio-demographic characteristics and the scores of depression about study sample (n=2080).

Variables	Baseline measure			Depression	
	Characteristic	N	%	Mean (SD)	p-value
Gender					*
	Male	1227	59	40.49 (5.82)	
	Female	853	41	41.04 (5.43)	
Age group (years)					
	18-30	441	21.2	40.41 (5.89)	
	31-40	849	40.8	40.86 (5.94)	
	41-50	609	29.3	40.71 (5.01)	
	51-68	181	8.7	40.85 (5.88)	
Ethnicity group					**
	Han	1674	80.5	40.93 (5.73)	
	Tibetan	147	7.1	39.80 (5.83)	
	Qiang	211	10.1	40.15 (5.08)	
	Hui	37	1.8	38.32 (5.14)	
	Others	11	0.5	39.63 (1.54)	
Education level					***
	Graduate	47	2.3	37.80 (8.01)	
	Bachelor	938	45.1	40.68 (6.10)	
	No degree	1095	52.6	40.88 (5.10)	
Monthly income (RMB)					***
	<1000	379	18.2	42.49 (6.17)	
	1000-2000	1347	64.8	42.32 (4.90)	
	2000-3000	274	13.2	40.60 (5.65)	
	>3000	80	3.8	39.67 (5.86)	

*** p<0.001; ** p<0.01; * p<0.05

Diagnoses and clinical characteristics were determined using the Self-Rating Anxiety Scale (SAS) and the Self-Rating Depression Scale (SDS) [17,18]. These are standard assessment instruments, which have been proved to accurately reflect the subjective feeling of patients with anxiety tendencies or depression severity, respectively. Both questionnaires have been validated and widely used in China with 20 items having been translated into Chinese. Each item had a 4-point Likert scale (0=never to 4= very often). Higher scores on the SAS or SDS are indicative of a higher level of mental disorder. According to the Chinese norm, when a score exceeded 50, it was determined that the person suffered from anxiety or depression. The internal consistency of the sub scales was good (*α*≥0.95).

Due to the high degree of similarity in the conceptual underpinnings of the personality variables, we ran a factor analysis to investigate whether social support was provided to survivors. Social support has been defined as ‘the assistance and protection given to the others, especially to individuals’ [19] measured by the Social Support Rating Scale (SSRS) [20], which has shown high reliability and validity on wide range of Chinese populations. On the basis of this exploratory factor analysis, 10 items on the scale were allocated to three subscales: subjective support, objective support and support availability. Each statement was scored on a 4-point Likert scale. The total score was used as a measure of the current total social support status, and the score for each category was derived from the scores for the corresponding items. The internal consistency of the scale was considered satisfactory (*α*>0.91).

### Procedure

Data collection was conducted one year after the Wenchuan earthquake. Before the formal investigation, we conducted a pilot test in May and June 2009 with a group of randomly selected survivors, after which we made minor modifications and adjustments to the survey instruments to ensure consistency and validity. Prior to the formal interview with the selected participants, there was a preliminary investigation to determine if any of these participants suffered from serious depression. Those that were found to have mental retardation or some other type of major psychoses (e.g. schizophrenia, major depressive disorder, organic mental disorders) were ineligible for the study. The final version of the questionnaire was used from July to September 2009. All assessment forms were translated from English to Chinese and back-translated by a bilingual team of professionals. 

As the areas affected by the earthquake each had different societal conditions, economies, populations and cultures, a stratified random sampling strategy was adopted by choosing interviewees randomly from temporary housing or tents. In the first sampling stage, 2300 participants were selected from two earthquake-stricken provinces, Sichuan and Shanxi, both of which included 19 heavily hit countries. In the second stage, we furthered stratified the sample by gender, age, education, ethnicity and monthly income. 

After a 5-day training program, our survey team was divided temporarily into different groups. Each had two psychology graduate students working as research assistants and a professional staff. After explaining the goals of the study, group members tried to word the questions as objectively as possible to make the participants’ own ratings less subjective. The items were essentially identical; however, sometimes fewer items were in the observed versions as some aspects could not be observed. As part of the informed consent process, participants were informed that their responses were confidential and they were free to withdraw from the study at any time. After completing a description of the total items, written informed consent was obtained. If a participant was unable to understand the questions, team members would assist them by giving a more detailed explanation. If a person declined the interview, the next closest inhabitant was invited. 

### Data Analysis

Various statistical methods were utilized in the study. First, list wise deletion was used for missing data. Then, descriptive statistics means and standard deviations were calculated using SPSS17.0 (SPSS Inc, Chicago, IL). All variables were treated as continuous. In detail, T-tests were conducted to examine the differences in the mean scores. Pearson correlations were performed to examine bivariate associations between the variables. To test potential moderators, regression analyses were used separately among several groups. After the F-test, we determined the standard equations from each step, which intuitively assisted us in recognizing the impact on depression. During the analysis, an effect was considered present if the resulting ρ-value was small (0.05 is usually deemed as the threshold).

## Results

### Demographics

The baseline demographic characteristics are presented in Table 1. The mean age of participants was 38.24 ± 8.82 years (ranging from 18 to 65 years). 59% of interviewees were male. Han were the majority ethnic group, with about 80.5% and the following ethnic groups; Tibetan (7.1%), Qiang (10.1%), Hui (1.8%), and others (like Tujia and Yi etc., 0.5%); also participated. Overall, 53% of respondents had a relatively low education level, and 83% earned less than 2000 RMB per month.

Grouped by demographic variables, the depression scores are also shown in Table 1. It can be seen that males reported lower scores than females, with a greater internal deviation (SD=5.82). Two of the age groups, especially those between 31 and 40 (mean=40.86, SD=5.94), had higher scores than the others. In terms of ethnicity, the national minorities scored less than the Han (mean=40.93). Those with a lower education level (mean=40.88, SD=5.1) and an income less than a 1000 RMB a month (mean=42.49, SD=6.17) had the highest scores. From the p-values, we can see that except for age, other demographic variables showed a significant relationship with depression.

After the significant omnibus test, post hoc analyses were conducted to determine the internal differences of five demographic categories with depression. In detail, different ages had no significance (p>0.05). For ethnicity, the relationship between Han and Zang (p=0.02), and Han and Hui (p=0.006) were significant. The level of education was significant except for the relationship between having no degree and having an undergraduate level qualification. The internal mean difference was not very significant for income.

### Correlation analysis

The correlation coefficients between the study variables are shown in Table 2, with the finding that the correlations between total social support, the three types of social support, and anxiety and depression were significant. The correlation coefficient between anxiety and depression was 0.67, which demonstrated that the relationship was notable. Social support and the three types of social support were also significantly correlated with each other positively, especially for that between objective support and support availability (r=0.555). Social support was negatively correlated with anxiety and depression as expected. Subjective support was found to have a greater impact on both anxiety and depression (r=-0.273, r=-0.328).

**Table 2:** Correlation coefficients among the study variables.

Variables	1	2	3	4	5	6
1. Anxiety	1.00					
2. Support	-0.278**	1.00				
3. SS	-0.273**	0.913**	1.00			
4. OS	-0.216**	0.752**	0.511**	1.00		
5. SA	-0.073**	0.501**	0.239**	0.555**	1.00	
6. Depression	0.670**	-0.303**	-0.328**	-0.179**	-0.089**	1.00
Mean	43.391	35.774	17.697	10.146	37.647	40.719
SD	7.835	6.164	4.139	2.314	3.288	5.666

SS subjective support, OS objective support, SA support availability

### Regression analysis

A regression analysis was conducted to investigate how the different types of social support influenced anxiety and depression. In order to decrease the possibility of multi-collinearity, the centralization of the variables was computed first. We can see how the three types of social support influenced anxiety and depression in Table 3. From this table we can conclude that the three types of social support were significant for anxiety, as it can be seen that they clearly influenced anxiety negatively. However, only subjective support was found to effect depression significantly. When it comes to demographics, age and education level were not significant for anxiety, but only age was found not to affect depression. Income was found to be negative for anxiety and depression. 

**Table 3:** Results of regression analyses.

Variables	Anxiety			Depression		
	SE	Beta	t	SE	Beta	t
Gender	0.334	0.078***	3.732	0.242	0.52*	2.473
Age group (years)	0.194	0.025	1.141	0.14	0.033	1.524
Ethnicity group	0.209	-0.063**	-3.065	0.151	-0.08***	-3.855
Education level	0.311	-0.003	-0.142	0.225	-0.051*	-2.347
Monthly income (RMB)	0.282	-0.212***	8.619	0.204	-0.093***	3.773
SS	0.045	-0.211***	8.801	0.033	-0.317***	13.229
OS	0.095	-0.117***	4.15	0.069	-0.007	0.237
SA	0.168	-0.152***	-5.525	0.121	-0.036	-1.312

A hierarchical multiple regression analysis was then conducted to investigate the size and effect of the moderator. The conclusions for the T-tests and F-tests are summarized in Table 4. In the first step of the model, a multiple regression analysis looking at anxiety with depression was conducted to examine the main effect, which indicated that anxiety had a significant influence on depression (p<0.001), and impacted depression positively (*β*=0.67). In Step 2, under the premise of controlling the dependent variable, the three types of social support were subjected to a regression analysis, with the finding that there was a significant effect in the ability of subjective support to predict depression (p<0.001), while objective support (p=0.641) and support availability (p=0.835) were not significant. Subjective support appears to decrease depression (*β*=-0.32). In Step 3, the interaction of anxiety and subjective support was added into the model as a predictor variable to assess the effect [21]. Finally, an equation was applied to express the relationship between depression and anxiety. The results showed that an adjustment in the social support, and especially in the subjective support for depression, strongly supported the social support buffering model.

**Table 4:** Results of multiple regression analyses (Depression as dependent variable) (N=2080).

Variables	Depression (Y)					
	Standard equation	F	R^2^	SE	Beta	t
Step 1	Y=0.67X	1695.97***	0.449			
Anxiety (X)				0.012	0.670***	41.182
Step 2	Y=−0.320M_1_	62.527***	0.108			
SS (M_1_)				0.033	-0.320***	13.225
OS (M_2_)				0.069	-0.013	0.466
SA (M_3_)				0.110	-0.005	0.208
Step 3	Y=0.672X−0.155M_1_+0.016XM_1_	619.283***	0.472			
Anxiety (X)				0.012	0.627***	37.85
SS (M_1_)				0.023	-0.155***	9.364
Anxiety × SS (XM_1_)				0.004	0.046*	2.118

Y the dependent variable depression; X the independent variable anxiety; M_1-3_ three types of social support.

We can see that anxiety plays an important role in the change in depression severity (R^2^=0.449, p<0.001). From Step 2, the equation showed that after the disaster, the greater an individual’s subjective support, the less effect anxiety had on the adverse effects of negative depression. However, the regulating effect of objective support and support availability were found to be not significant. Finally, the interaction between subjective support and anxiety was found to make a significant contribution to the level of depression (R^2^=0.472, p<0.001), which demonstrated a positive moderation between anxiety and depression, further indicating that social support has a negative effect. Thus, the hypotheses were supported by this analysis, which proved that social support has a negative effect on the level of depression. On the whole, the study proved that not all interactions between social support and demographic factors impact every domain of anxiety or depression.

## Discussion

In this study, we examined the function of social support in moderating the relationship between anxiety and depression after interviewing 2080 Wenchuan earthquake survivors one year after the event. As predicted, it was demonstrated that social support mitigates the negative effects of anxiety or depression amongst earthquake survivors, showing that anxiety was strongly and positively related to depression, while social support weakened depression. The main results can be catalogued as follows. Firstly, except for age, most demographic factors showed a significant relationship with depression. Secondly, anxiety was shown to have a high positive impact on depression, meaning those with high anxiety were more likely to become more easily depressed. Thirdly, social support was found to contribute to a significant change in the anxiety-depression model. In particular, subjective support was proved to negatively moderate depression. The data corroborated the hypothesis that anxiety and depression are the result of a multivariate model of a combination of socio-demographic influences, and social support, which is consistent with Glazier et al. [22]. Interestingly, although we found social support moderated the relationship, the observed pattern deviated from our predictions in that only subjective support had a strong effect on depression. 

Social support was negatively correlated with anxiety and depression, in accordance with Potochnick, in that lower levels of social support were associated with greater increases in depressive symptoms [23]. This could be explained in that increased social support means a greater chance to have contact with others and more abreact ways to help survivors avoid staying alone in deep entertain foolish ideas. To avoid mental disorders such as depression after earthquakes, social support is very necessary. From the regression analysis, among the interaction variables, subjective support was seen to be an important factor in the prevention of depression severity, which is consistent with the hypothesis that a moderator variable can influence people’s behavior [24-26]. Consequently, one main explanation for this situation could be that people with a high level of subjective support tend to use more effective coping strategies than those with a lower level. Thus, they naturally prevent themselves from feeling avoidance, emotional numbing and arousal, and as a result, were more active and efficient in social activities, and had fewer difficulties in their daily live. However, Blazer and Hughes revealed that subjective support and depressive symptoms are interrelated but separate constructs [27].

In this study, objective support and support availability were not found to be significant for depression. However, Cheng et al. demonstrated that objective support was significant for a patients’ degree of depression [28]. This situation could be explained by the fact that as the survey was only taken one year after the earthquake, survivors would still be feeling a significant sense of loss and would have less emotion towards needing material goods, so direct assistance would not seem so urgent at that time. Several studies have also implied that support availability is an important factor influencing the psychological environment of disaster victims, which in this study was shown to have nothing to do with the negative effects of anxiety or depression. This was consistent with conclusions from previous studies, which stated that different types of social support may play different roles in health [29]. However, in the long run, people who received less social support tended to show continued depression [30]. This reminds that different levels of social support should provide to people in time.

In addition, the study also found that certain demographic characteristics were closely related with depression. This situation can be explained by the fact that females tend to be more emotional and tended to be more affected by disaster and its consequences, so as a result were more likely to suffer depression than men. This conclusion has also been demonstrated in previous findings [31,32]. Age, however, was not a predictor for depression, which is also in accordance with previous studies [33]. This may be because younger people were less sensitive towards the disaster than older people and therefore could not fully realize the scale of the tragedy. Middle-aged people, and especially those the age range of 31-40 with children, tended to be the family members who carried the greatest burden and therefore were more likely to show depression. Older adults showed a higher prevalence of anxiety and depression, which is consistent with Ritchie et al. [34]. In terms of ethnic groups, an interesting finding was that the Han group had higher social support scores and displayed more depression. The probable cause is that most minority ethnic groups in China share a religion and faith and have a high happiness index, while very few people in the Han ethnic group are religious. When the Han have disorders, they are often unable to find a way to release this stress, which leads them to conceal the stress and consequently develop mental disorders. This finding, however, does not agree with that of Dirkzwager et al. [35]. With regard to education and income, survivors with a lower education and income were more likely to show depression. More highly educated people tend to have a much wider field of vision and greater world experience, so they are often more able to adjust over time. Survivors with a lower income worried more about their daily life, which is consistent with a previous study which showed that a lower household income was related to a higher psychological morbidity [31].

The findings of this study have been described above. However, there are several limitations to the study. Firstly, there is a lack of data from the non-disaster areas, which could result in some deficiencies in this study. Secondly, during the survey, most adults went to work leaving the old and the young at home, which means that the ages in this study may not reflect the anxiety tendencies of all ages and the family status information was not considered. Thirdly, a return visit has not yet taken place, thus a longitudinal study is not available, but we are preparing for it at present. Another limitation of this study is that self-report instruments were used, so answers may be somewhat subjective and may not reveal the true situation, with some participants being over or under reported. 

Keeping these limitations in mind, some implications to assist disaster survivors and avoid psychological disorders and improve mental health are outlined. Though several years have passed since the earthquake, the effects may still linger. Attention to the stricken areas should be continuous, not only in the provision of supplies, but also with a focus on the mental health of the survivors. The results of this study emphasize that social support, as a moderator, is important to decrease anxiety and depression. Humanistic concerns such as early identification, ongoing monitoring, and sustained psychosocial support should be offered. More objective support and support availability from government and society should be paid to females, low-income families and those with a lower level of education. The Han ethic group should enhance their cohesion and faith to overcome anxiety or depression.
